# New Fusible Alloys

**Published:** 1860-07

**Authors:** B. Wood

**Affiliations:** Nashville, Tenn.


### DENTAL REGISTER OF THE WEST.

VOL. XIV.] JULY, 1860. [No. 1.

### Original Essays and Communications.

### NEW FUSIBLE ALLOYS.

BY B. WOOD M. D.

In availing myself of this opportunity to furnish you with particulars as to the advantages claimed for the Metallic Composition, for which a patent has lately been issued to me, it will be necessary to advert briefly to some of the alloys upon which mine is offered as an improvement.

I may premise by stating that, having instituted a series of experiments with a view to the production of alloys, possessing great fusibility in connection with tenacity, malleability and other qualities required of a metal, as a solder for the more fusible metallic wares in use, and as a material for casting at a low temperature, &c., and having discovered in the metal cadmium as an ingredient in alloys, properties favorable to the result, I attained the object sought by combining this metal with lead and tin, and with lead, tin and bismuth, in the proportions as set forth in my specifications: —to these mercury may be added to modify the result for particular cases as described.

The advantage of possessing the joint qualities of great fusibility, malleability, strength, &c., in a metal designed for use as above, is too evident to dwell upon. Much patient experimentation has been conducted towards this end, and and the research has been rewarded by the discovery of many valuable alloys.

One of the most useful of this class is the alloy commonly called "fine solder," consisting of one part of lead, and two parts tin. It is perfectly malleable, highly tenacious, and melts according to Prof. Graham, at the temperature of 360 deg. Fahr., being the most fusible of any of the mixtures of lead and tin. But its melting point is too high for a solder for the more fusible tin-metals, such as the ordinary pewter and Britannia ware, &c. Another objection is its softness.

The alloys consisting of lead, tin and bismuth, commonly called "bismuth solders," are harder and more fusible, but they are proportionably brittle. A common formula for very easily melted solder is 16 parts tin, 8 lead, 4 bismuth. A more fusible mixture and the most fusible *alloy* hitherto known is that discovered by Sir Isaac Newton, consisting of 3 parts tin, 5 lead, 8 bismuth. This melts, according to Prof. Graham, at 202 deg. Fahr. No practical improvement has ever been made upon this by any combination of the constituents, although certain combinations possess, according to some experimenters, a lower melting point by one or two degrees—a difference too slight for appreciation by ordinary tests. Practically the melting point is somewhat higher, requiring a temperature of about 210 deg. for perfect liquifaction. In view of its remarkable fusibility this alloy has received the distinguished name of "Fusible Metal." It is too brittle for ordinary use as a solder, but is much employed for casting, as in making dies for light work, and for taking impressions, from medallions, and other objects. Melting below the temperature of boiling water, it may be used upon the fresh plaster cast, or other moist surface. But it has the disadvantage that when used at a heat barely sufficient for fusion, it is not fluid enough to take the sharp outlines, and congeals before it can flow into the interstices; while a small additional heat raises its temperature above that at which water boils, whence steam is produced which spoils the work.

The melting point of these alloys may, as is well known be lowered to any extent by the addition of mercury, but this metal even in small proportions renders them so frail, and brittle as to be worthless for the ordinary uses. It also causes them to tarnish, and is partly eliminated from the compound, being retained rather as a foreign admixture than as a chemical constituent, whence it occurs that these *amalgams* injure other metals with which they come in contact. So also when used for anatomical injections the mercury permeates, and blackens the tissues.

My improvement greatly obviates these defects and meets more perfectly the requirements of alloys of this class. The composition composed of cadmium, lead and tin, melts somewhat under 300 deg. Farh., or sixty or seventy degrees below the melting point of the "fine solder" above referred to. It is equal to it in malleability and tenacity, is much harder and stronger, and admits of a higher polish. It ranks in fusibility with the more easily melted "bismuth solders," and is believed to be greatly superior to any of them in all the other requisites for this purpose. The advantages for other purposes of a metal possessing these qualities will readily suggest themselves.

The composition consisting of cadmium, lead and tin, in conjunction with bismuth, melts between 150 and 160 deg. Fahr., being some fifty or sixty degrees below the melting point of Newton's "Fusible Metal," mentioned above, corresponding very nearly with it in respect to malleability and tenacity, but harder and more adhesive. It is adapted to similar purposes, and the low temperature at which it fuses renders it applicable in many cases where the other would not answer, while it is free from the objections appertaining to the amalgams resorted to in such cases. As a material for anatomical injections it will be found superior in every respect, it is believed, to the amalgams in use.

This alloy, it will be observed, is much more fusible than any other *alloy* known in the arts, or than any combination of metals whatever except amalgams. I speak here of the permanent metals without regard to what possibly may have been produced in the chemist's laboratory by combinations of such unstable metals as potassium, and sodium, which, it is evident, would not be permanent in ordinary conditions, much less be of practical application in the arts.

Both of these forms of alloy are susceptible of considerable modification as to their physical characters, such as hardness, rigidity, pliancy, &c., by varying the proportions of their constituents, as set forth in my specification.

Their melting point may be lowered, like that of other alloys, by the addition of mercury, but what is peculiar, this metal may be employed to a certain extent in connection with them without materially impairing their tenacity, whereas, if used in like quantity in any analogous alloy not containing cadmium, it would render the compound so frail and brittle as to be worthless. Moreover, cadmium having a strong affinity for mercury, and affording a bond of chemical union between it and the other constituents, a true chemical combination appears to result:—whence the presence of mercury is less objectionable than in the cases before referred to.

From the results of my experiments I doubt not that cadmium, which has so long been passed over as of little practical utility, is destined to become a very important metal in the arts. It is usually found associated with zinc. If the reports be true that zinc has been found extensively in the Western States, it is possible cadmium may be obtained in connection with it in greater abundance than heretofore produced from other localities, and it might be worth the while of our metallurgists to direct their attention to the subject.

NASHVILLE, TENN., April 28, 1860.

